# Effects of ceftiofur treatment on the susceptibility of commensal porcine *E.coli* – comparison between treated and untreated animals housed in the same stable

**DOI:** 10.1186/s12917-015-0578-3

**Published:** 2015-10-15

**Authors:** Anne Beyer, Sven Baumann, Gesine Scherz, Jessica Stahl, Martin von Bergen, Anika Friese, Uwe Roesler, Manfred Kietzmann, Walther Honscha

**Affiliations:** University Leipzig, Faculty of Veterinary Medicine, Institute of Pharmacology, Pharmacy and Toxicology, Leipzig, Germany; Helmholtz Centre for Environmental Research – UFZ, Department of Metabolomics, Leipzig, Germany; University Leipzig, Faculty of Biosciences, Pharmacy and Psychology, Institute of Pharmacy, Leipzig, Germany; University of Veterinary Medicine Hannover Foundation, Institute of Pharmacology, Toxicology and Pharmacy, Hannover, Germany; Helmholtz Centre for Environmental Research – UFZ, Department of Proteomics, Leipzig, Germany; Department of Biotechnology, Aalborg University, Chemistry and Environmental Engineering, Aalborg, Denmark; Free University Berlin, Institute for Animal Hygiene and Environmental Health, Berlin, Germany

**Keywords:** Cephalosporins, ESBLs, Swine, Stable dust, Aerosol

## Abstract

**Background:**

Healthy farm animals have been found to act as a reservoir of extended-spectrum β-lactamase (ESBL)-producing *Escherichia coli (E. coli).* Therefore, the objective of the study was to determine the input of antimicrobial active ceftiofur metabolites in the stable via faeces and urine after intramuscular administration of the drug to pigs and the elucidation of the *Escherichia coli* ESBL resistance pattern of treated and untreated pigs housed in the same barn during therapy.

**Methods:**

For determination of the minimal inhibitory concentration (MIC) the method of microdilutionaccording to the recommended procedure of the Clinical and Laboratory Standards Institute was used. Inaddition to that, a qualitative determination was performed by agar dilution. Unsusceptible E. coli speciesselected via agar dilution with cefotaxime were confirmed by MALDI-TOF and ESBL encoding genes wereidentified by PCR.

The amounts of ceftiofur measured as desfuroylceftiofur (DFC) in the different probes (plasma, urine, faeces and dust) were analysed by UPLC-MS/MS.

**Results:**

In a first experiment two groups of pigs (6 animals per group) were housed in the same barn in two separated boxes. One group (group B) were treated with ceftiofur according to the licence (3 mg/kg administered intramuscularly (i.m.) on three consecutive days, day 1–3). During a second treatment period (day 29–31) an increased rate of ESBL resistant *E. coli* was detectable in these treated pigs and in the air of the stable. Moreover, the second group of animals (group A) formerly untreated but housed for the whole period in the same stable as the treated animals revealed increased resistance rates during their first treatment (day 45–47) with ceftiofur. In order to investigate the environmental input of ceftiofur during therapy and to simulate oral uptake of ceftiofur residues from the air of the stable a second set of experiments were performed. Pigs (6 animals) were treated with an interval of 2 weeks for 3 days with different doses of ceftiofur (3 mg/kg, 1 mg/kg and 0.3 mg/kg i.m.) as well as with 3 mg/kg per os) and the renal and biliary excretion of ceftiofur as its active metabolite were measured in comparison to the plasma levels. In addition to that, probes of the sedimentation dust and the air of the stable were analysed for drug residues.

**Conclusion:**

The present study shows that treatment of several animals in a stable with ceftiofur influences the resistance pattern of intestinal *Escherichia coli* of the treated as well as untreated animals housed in the same stable. During therapy with the drug which was administered by injection according to the licence we detected nameable amounts of ceftiofur and its active metabolites in the dust and air of the stable.

**Electronic supplementary material:**

The online version of this article (doi:10.1186/s12917-015-0578-3) contains supplementary material, which is available to authorized users.

## Background

Antimicrobial resistance is a worldwide serious problem in human and veterinary medicine. Therefore a series of World Health Assembly resolutions [1] have been adopted in order to enforce the development of the WHO global strategy 2001 [2], the EU Antimicrobial Resistance Strategic Action plan (2011) [3] and the EU Council Conclusions (2012) [4]. As a result of these activities a lot of studies were initiated concerning the development, spread and occurrence of bacterial resistance in human and veterinary medicine. Recently healthy farm animals have been found to be a reservoir of extended-spectrum β-lactamase (ESBL)-producing *Escherichia coli (E. coli)* with a prevalence of 10.7 to 36.3 % in pigs [5]. Such isolates have been found in farm animals and pets in different countries [5–9]. From farm animals the most common type of ESBL gene of *E. coli* belongs to the CTX-M group conferring resistance to third- and fourth-generation cephalosporins [10, 11]. Indeed, it has been shown that ceftiofur and cefquinome exerted selective effects beyond the withdrawal times for CTX-resistant coliforms due to proliferation of indigenous CTX-M producing strains and by horizontal gene transfer [12]. Due to the horizontal transfer by plasmids, transposons and integrons resistance genes could be exchanged very fast and between different bacterial species [13]. Thus, ESBL-producing *E. coli* are held to be responsible for the broad occurrence [14] and for severer courses of many diseases [15, 16]. In recent years several studies were published in which the application of antibiotics in livestocks cause the increase of resistance [17, 18]. Thus, an enhancement of selective pressure can influence the situation of resistance [19–21] and strengthens the development and dissemination of resistance in livestock populations [22].

In addition to the impact of antimicrobials on the microbiome of treated animals the usage of antibiotics applied with feed involves a high risk of environmental pollution induced by drug residues. A study performed by ZESSEL [23] revealed concentrations of sulfadiazin up to 1.9 μg/mg in stable dust depending on position of collecting and feeding formulation. This environmental input of antimicrobials can be incorporated by animals standing in the same stable with the result of detectable drug levels in plasma and urine [23, 24]. But not only the application of drugs via feed is an explanation for the occurrence of antimicrobial residues in the environment, the study of SCHERZ et al. [25] revealed that the drug and its metabolites are even present in sedimentation dust and aerosol becoming bioavailable via excrements of the treated animals.

Ceftiofur is a third-generation cephalosporin licensed in Germany for the treatment of bacterial respiratory diseases in swine and cattle as well as of interdigital necrobacillosis and postpartum metritis in cattle [26]. After intramuscular application ceftiofur is quickly metabolized to desfuroylceftiofur (DFC) by elimination of furoic acid [27, 28]. Due to the integrity of the β-lactam ring this metabolite still retains antibacterial activity [29]. DFC rapidly forms conjugates with plasma and tissue proteins [30] or is metabolized to disulfides like DFC-gluathione disulfide, DFC-cysteine disulfide or DFC-Dimer [29]. All conjugates can be deconjugated to DFC and therefore recover antimicrobial activity [31].

The aim of the study was to investigate the input of ceftiofur in the stable after intramuscular injection of the drug and the observation of the resistance situation of the gut microbiome of swine. Furthermore occurrence of ESBL-producing *E. coli* after an approved treatment with ceftiofur and the influence on untreated animals of the same barn was investigated. For this the transfer of resistant bacteria or the exposure to minimal concentrations of the active substance originating from the excretion of treated pigs were considered.

## Methods

### Animals

At an age of four weeks healthy pigs (weight range from 8 to 9.2 kg) were kept after random allocation to two groups (each of six) in a stable for the first part of the experiment (experiment I) in which the development of resistant *E. coli* in treated and non-treated animals was investigated. The animals were fed with piglet starter food and had free access to water. Before starting the experiment rectal swab samples were taken from each animal and the presence of ESBL resistant *E. coli* were checked in the gut microbiome of the animals by an enrichment-procedure and agar dilution. In the second part of the experiment (experiment II) six female pigs weighing 10 to 11 kg were used.

All animal studies were conducted according to institutional guidelines for ethical care and use of animals for experimental and other scientific purposes. The study was registered by the Lower Saxony State Office for Consumer Protection and Food Safety (registry number: 33.12-42502-0-11/0338, Lower Saxony State Office for Consumer Protection and Food Safety). The registration procedure contains an approval of an internal ethic committee.

### Experimental design

In the first part of the experiment the influence of ceftiofur on the intestinal microbiota of the pigs was investigated. For the experiments a barn with dimensions of 4.8 m × 1.9 m was divided in two boxes via a middle corridor (width 1.60 m). As result of this areal separation a direct contact of the grouped animals was excluded. To minimize points of contact between both animal groups, each box had its own stable equipment for cleaning and feeding. Protective clothing consisting of one-way overalls, gloves and over boots were used in addition to the working clothes for each entry in the stable. Afterwards the one-way articles were depolluted. To avoid a possible cross-contamination the handling and maintenance of the animals always start with the control group (group A).

The first group of animals (group A) served as antimicrobial-free control group and was not treated until day 45 of the experiment. To study the influence of a therapeutic dosage on the physiological enteric microbiota the swine of the second group (group B) were treated with 3 mg/kg/day ceftiofur hydrochloride on three consecutive days (day 1 to 3) and for a second time on days 29 to 31. For investigation of a possible impact of an antibiotic application on pre-exposed but formerly untreated animals the animals of the control group (group A) were treated on day 45 to 47 for the first time. Probes (faeces, dust and aerosol probes for MIC determination) were collected on the following days: 0, 5, 8, 14, 21, 28, 34, 37, 42, 50, and 53.

For collecting of dust of the stable filter pumps were used and in addition to that, samples were drawn by agar plates to observe the resistance situation of the environmental *E. coli* in the stable. Two of these pumps (flow range between 2.7 L/min and 3.625 L/min ) with sterile polycarbonate filters were placed at a distance of 30 cm from the treated group (group B) at the middle corridor. After running for 1 hour filter membranes from the pumps were transferred into 5 mL of Fluorocult®-LMX-broth (LMX-broth modified acc. to Manafi and Ossmer; Merck, Darmstadt, Germany) using a sterile tweezer. In addition two opened endo-agar plates (lactose-fuchsin-sulfite agar, Merck) were set on the brink of each box in the stable for 1 hour (height 1 m).

For the second part of the experiment (experiment II), only one box of the stable was used for six pigs. These experiments were performed in order to elucidate the input of ceftiofur residues into the stable due to biliary and renal excretion of animals treated with different dosage regimes. In addition, oral uptake of ceftiofur residues via air was simulated by a per os treatment with 3 mg/kg ceftiofur. To measure the excretion of DFC and its metabolites animals (*n* = 6) were treated in an interval of 2 weeks each time with different doses of ceftiofur (3 mg/kg, 1 mg/kg, and 0.3 mg/kg) i.m. as well as 3 mg/kg p.o. via feed three times every 24 h. For per os treatment the amount of ceftiofur was added to some sugar cubes and given to the animals.

Urine and faeces were collected separately on different days (day 0, 1–3, and 7, 9, 11). These samples were spontaneous excreted and placed into plastic tubes. In addition to that, sedimentation dust was collected from five different positions of the stable by using brand-new playing cards at the same days (see above). Four of these positions were close to the treated animals at the window ledge (position 1), partition grid (positions 2 and 3), and the ground beside the box (position 4). A distance of 1.60 m was between collecting position five and the box of the animals. To collect aerosols filter pumps were used for 8 h per sampling day by using polycarbonate as filter material. They were arranged at two positions close to the box. Dust and filters were placed into glass vials and covered with metal foil. All samples were stored light-protected at −20 °C until preparation.

Plasma samples of each treatment cycle were obtained directly before and at various time intervals after the injection of ceftiofur via syringe from the jugular vein. After i.m. injection blood samples were taken at 0, 24, and 48 h, whereas blood samples after per os application were collected at 0, 2, 4, 6, 24, 48, 50, 52, 54, 71, and 96 h. After centrifugation at 4 °C (3000 x g) for 10 min plasma was collected from blood samples and stored at −80 °C.

### Antibiotics

Ceftiofur (ceftiofur hydrochloride, ready to use product; Vétoquinol) was administered by intramuscular (IM) injection in the musculus trapezius of the neck. For measuring the concentration of DFC and DFC-metabolites in plasma ceftiofur sodium (ceftiofur sodium, sterile powder; Excenel®) was applicated.

### Isolation and MIC-determination of non-type-specific *E.coli*

The detection of the minimal inhibitory concentration (MIC) followed the “performance standards for antimicrobial disk and dilution susceptibility tests for bacteria isolated from Animals”-M31-A2 of the Clinical and Laboratory Standards Institute [32]. The method of microdilution was used. Microtiterplates containing serial dilutions of ceftiofur concentrations from 0.125 μg/mL up to 64 μg/mL (final concentration after inoculation) were made in the laboratory and stored in a freezer at −70 °C. Mueller-Hinton II broth was used for dilution.

Within 1 hour after sampling 1 g of faeces were homogenized in 9 mL of sterile physiological sodium-chloride solution, streaked onto endo-agar plates and incubated for 24 h at 37 °C.

For the examination of susceptibility, subcultures of at least ten single colonies, morphological typical for *E. coli,* were sub-cultured on Mueller-Hinton-agar. The susceptibility tests were always performed with colonies from overnight plates. An inoculum of 5 × 10^5^ colony forming units (CFU)/mL per well, adjusted by densiotometry to 0.5 McFarland turbidity standard with Mueller-Hinton-II-broth, was transferred to microdilution plates. To protect the filled trays against dehydration during incubation at 37 °C for 20–24 h the plates were covered with an adhesive foil [33]. For a positive control the reference strain ATCC 25922 was used and MICs were compared to the required MIC reference range (0.25 μg/mL to 1 μg/mL). Furthermore, a control for growth and a negative control were conducted for each colony. To determine CFU’s in inoculum suspensions a dilution of each applied inoculum was plated on Cystine-Lactose-Electrolyte Deficient (CLED)-agar. A number of 20–80 CFU [33] was set as reference range. The purity of the inoculum was currently checked. To confirm the presence of *E. coli* in the examined samples an aliquot of each CFU was transferred into Fluorocult® LMX Broth. A color change of the broth from yellow to blue, a blue fluorescence under long-wave UV light and a positive indole reaction with Kovacs reagent was regarded as highly specific for *E. coli*. Only bacterial colonies which fulfilled all these criteria were further evaluated and the MIC-values determined.

In addition to a quantitative examination with microdilution a qualitative determination was performed by agar dilution. A 1:10 dilution in Lysogeny broth LB-medium of each fecal sample and sampling day was incubated at 37 °C for 24 h and 10 μL of the culture was plated on endo-agar plates containing certain ceftiofur concentrations (0 μg/mL, 1 μg/mL, 2 μg/mL, 8 μg/mL). For confirmation of *E. coli* Fluorocult LMX broth in combination with Kovacs reagent was used.

### Verification of ESBL producing *E. coli*

After selection of unsusceptible *E. coli* via agar dilution with cefotaxime containing MacConkey-agar (1 μg/mL), the species was confirmed using MALDI-TOF identification (MALDI Microflex ® LT and Biotyper ® database, Bruker Daltonics, Bremen, Germany). Identification of ESBL encoding genes of the CTX-M, TEM, SHV and CYM families was carried out by PCR according to the method published by Roschanski et al. [34].

### Isolation and detection of *E. coli* in the environment

At the same day of sampling (see description of experiment I) uncovered endo-agar plates were placed in the stable. After 1 hour these plates were closed and incubated for 24 h at 37 °C. Five colonies per plate were selected and MIC’s determined by microdilution.

The filter membranes of the filter pumps were transferred in Fluorocult LMX broth and following an enrichment of the bacteria at 37 °C for 24 h the growth of *E. coli* was examined by color change, blue fluorescence and positive indole reaction. In the case of growth 10 μL of the suspension were plated on endo-agar containing different concentrations of ceftiofur (0 μg/mL, 0.5 μg/mL, 1 μg/mL, 2 μg/mL, 8 μg/mL). After incubation (37 °C for 24 h) the growth of CFU of *E. coli* was evaluated.

### Analysis of desfuroylceftiofur (DFC) in the environment

#### Pretreatment of samples

To avoid excessive amounts of DFC all urine samples of the dosing days (1 to 3) were diluted 1:100.

To 1 mL of urine, 150 mg faeces, 4 mg dust and the filter from the air pumps 7 mL extraction solution [sodiumtetraborat-decahydrat (0.95 % *w/v*), dithiothreitol (0.23 % *w/v*)] were added. The samples were vortexed and placed in a water bath at 50 °C for 15 min. Each 3 min the tubes were inverted. Subsequently after adding of 1.5 mL derivatization solution [sodiumhydroxid solution (3.5 % *w/v*), iodoacetamid (5 % *w/v*)] to the sample, the dilutions were vortexed again and mixed gently in the dark at room temperature for 1 h. After adding of 250 μL acetic acid in water (20 % *v/v*) the samples were centrifuged for 10 min (4 °C, 3000 × g).

For solid phase extraction (SPE) all cartridges were cleaned, preconditioned with 2 mL of a solution of acetonitrile in water (1:1) and afterwards preconditioned using 2 mL bidest water. The following extraction protocol was used: the whole supernatant was applied to SPE columns and unbound sample components removed by washing with 3 mL bidest. After drying the column by applying vacuum for 15 s, the analytes were eluted using 1.5 mL of a solution of acetonitrile and bidest (1:1) into a test tube. The eluate was concentrated to a volume of 0.5 mL via SpeedVac® (Eppendorf, Hamburg, Germany), transferred to an autosampler vial and finally filled up to a volume of 1 mL with bidest.

The calibration of DFC was accomplished by pretreatment of ceftiofur sodium (Excenel®) according to the above mentioned protocol. For each specimen (plasma, urine, dust and faeces) matrix matched calibrations were prepared.

### Analytical method

The amounts of DFC were analyzed with ultra-performance liquid chromatography (UPLC) in combination with tandem mass spectrometric detection (MS/MS). All detections were performed on an UltiMate 3000 RSLC (Thermo Fisher Scientific, Dreieich, Germany) coupled to a 5500 QTRAP mass spectrometer (AB Sciex, Darmstadt, Germany) equipped with an ESI Turbo Ion Spray source. For separation by liquid chromatography a Acquity BEH C18 separation column, (50 mm × 2.1 mm, 1.7 μm, Waters) connected to a VanGuard BEH Shield RP18 (1.7 μm) pre-column at 60 °C was used in gradient elution mode. The mobile phase consisted of eluent A (water, 0.1 % formic acid) and eluent B (acetonitrile, 0.1 % formic acid) at a flow rate of 900 μL/min. The gradient of Eluent B was: 0 min 5 %; 0 min 5 %; 1.5 min 20 %; 1.6 min 50 %; 2.1 min 50 %; 2.2 min 5 %; 3 min 5 %. The injection volume of each sample amounts to 5 μL.

For DFC quantification mass spectral data were acquired in positive ion electrospray ionization (ESI) mode using the multiple reaction monitoring (MRM) scan mode. Optimized ESI-MS/MS-dependent parameters were as following: ion spray voltage 5.5 kV, ion source gas 1, ion source gas 2, curtain gas, collision gas 40, 60, 20, medium (arbitrary units), respectively. The ion source was run at a temperature of 600 °C. The precursor ion for DFC was the ([M + H]+) ion of m/z 487.0. Characteristic product ions were at m/z 241.0, 227.0, and 210.0 completing the MRM-transitions for DFC quantification. Declustering potential, entrance potential and collision cell exit potential experiments were optimized and set to 120 V, 7 V, 13 V, respectively. By using weighted (1/x) least-squares regression analysis the calibration curves were obtained.

### Statistical analysis

Results of the MIC determination by microdilution were analysed with Exact Fischer Test using Microsoft Excel software (Office 2007). Statistical significance was assumed at p-values of ≤ 0.05.

## Results

### Microbiological studies (experiment I)

For discrimination between susceptible or resistant *E. coli* the epidemiological cut-off (Ecoff) and the clinical breakpoint (cbp) were used. For *E. coli* and ceftiofur the Ecoff was set to ≤ 1 mg/L [35]. Both EUCAST and CLSI could not fix a clinical breakpoint for ceftiofur and swine. Nevertheless for cow the a cbp of ≥ 8 mg/L was published by the CLSI in the “Approved Standard M31-A2” document [32] and was adopted in place for swine in this experiment.

#### Isolation and detection of *E.coli* in swine

**Fig. 1:**
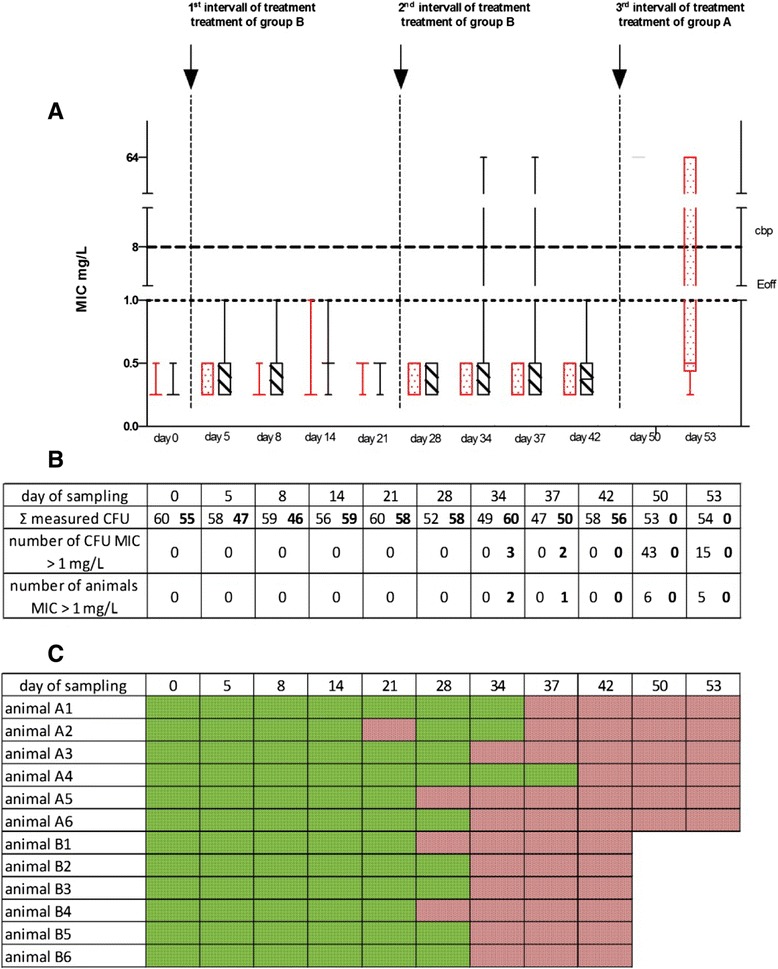
Results of the microbiological studies (experiment I). **a** Results of microdilution assay, detection of MIC-values of 10 *E. coli* colonies per animal and sampling day (left red Boxplot: group A (*n* = 6 animals), right black boxplot: group B, *n* = 6 animals) after treatment of pigs; Ecoff: epidemiological cut-off (≤1 mg/L) ; cbp: clinical breakpoint (≥8 mg/L). **b** Overview about the number of examined CFU, number of detected CFU with MIC >1 mg Ceftiofur/L and number of swine with increased MICs. Starting at day 34 the numbers on the left side of each field in the table stands for the value of group A, whereas the second value stands for group B. **c** Results of agardilution assay; green box: MIC < 1 mg Ceftiofur/L, red box: MIC > 1 mg/L

On the day before ceftiofur was applicated for the first time (day 0) only MICs below the Ecoff were determined as expected. Up to day 28 exclusively CFUs with MICs < 1 mg/L were detected by microdilution without initial enrichment of the bacterial colonies in the samples of all animals after the first treatment of the animals of group B (Fig. 1a and b).

The results of both groups (untreated and treated) did not differ and demonstrated the presence of the wild type of *E. coli*. After the second treatment of group B the results between both groups differed. While in the samples of animals from the untreated group (group A) no resistant CFUs were found, two animals of the treated group showed three *E. coli* isolates with MICs > 64 mg/L on day 34 (Fig. 1b). At the following sampling day (day 37) two colonies with an increased MIC could be found in the samples from this group of animals. These *E. coli* differed from the wild type and could be declared as resistant from an epidemiological standpoint. On day 42 none *E. coli* with increased MICs were found by microdilution.

Subsequent to the second treatment of group B, the previously untreated animals of group A received ceftiofur (3 mg/kg i.m.) according to the market authorization for the drug on day 45 to 47. In the fecal samples on day 50 of all 6 animals of this group 43 resistant CFUs in total were determined. On day 53 the number of measured CFUs, with an increased MICs dropped to 15 CFUs (Fig. 1b). Considering the results of microdilution a statistically significant difference (p < 0.001) was observed between the number of resistant CFUs in the samples from the treated group B after its first and second treatment compared to group A after its treatment.

The simultaneously performed agar dilution assay (Fig. 1c) revealed in none of the fecal samples *E. coli* with an increased MIC (>1 mg/L) on day 0 of the experiment and confirmed the absence of resistant *E. coli* in the gut microbiome of the animals in both groups. The same results were received for the days 5, 8 and 14. On day 21 with the aid of an enrichment procedure in one swine of group A *E. coli* with a MIC of > 2 mg/L (but < 8 mg/L) were detected. The presence of resistant *E. coli* in this animal could not be demonstrated on the following days of sampling (day 28 and 34). Resistant *E. coli* (>1 mg/L) were found on day 28 in two fecal samples of group B and in one of group A. The presence of non-wild type *E. coli* was demonstrated on day 34 in all samples of the treated group and in three of the other group after the second treatment of group B. Resistant *E. coli* were found in the fecal samples of all animals on day 42. In these cefotaxim resistant bacteria the genes of the enzyme families TEM and CTX-M were detected by PCR (data not shown). Occurrence of the TEM genotype does not confirm the presence of ESBL because not every derivate of this enzyme is responsible for resistance to oximino-chepalosporins whereas all enzymes of the CTX-M family possess the ESBL phenotype.

#### Isolation and detection of *E.coli* in the environment

**Fig. 2:**
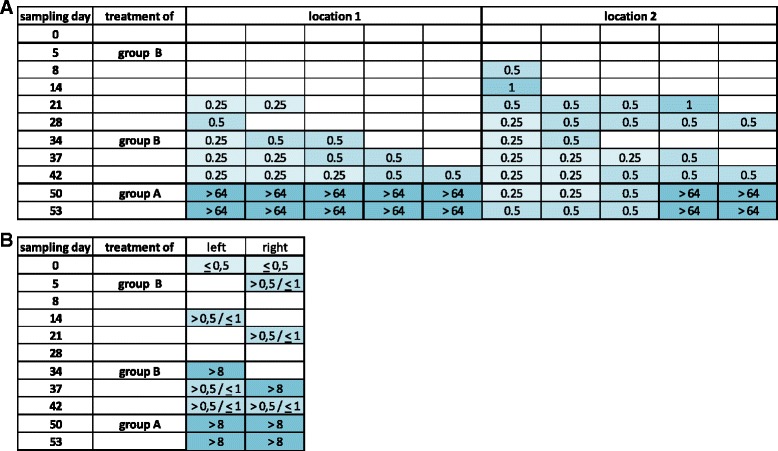
MIC-values of *E. coli* isolates collected by endo-agar plates by sedimentation (**a**) and by air pumps (**b**) in the stable after treatment of 6 animals of each group with 3 mg/kg b.w. i.m. (experiment I). **a** Determination of MIC-values from single colonies (*n* = 5) isolated from uncovered endo-agar plates via microdilution. Endo-agar plate 1 (location 1) was located in front of box A, whereas the second agar plate (location 2) was positioned besides box B. **b** MIC-values of *E. coli* isolates form the air of the stable after enrichment and agardilution. Left air pump was positioned at the side of box A and the second air pump was located close to box B

At the beginning of the experiment it was not always possible to isolate *E. coli* from the environment either by endo-agar plates or by air pumps (Fig. 2).

By using endo-agar plates (Fig. 2a) the first bacterial colonies were obtained on day 8 (*n* = 1) and day 14 (*n* = 1) of the experiment from the area of the animals of group B (location 2 of the endo-agar plate) and on day 21 from area of group A (*n* = 2, location 1). In the course of the experiment the number of *E. coli* CFUs increased. Within the interval between the first and the second treatment of the animals of group B 3 to 5 CFUs per sampling day and location were detected. In this timeframe all MICs of these *E. coli* were below the Ecoff, with the exception of one colony on day 14 and 21 each in the area of the treated animal group. After the treatment of the formerly untreated group A (treatment days 45 to 47) *E. coli* with increased MICs (≥64 mg/L) were recovered. In the location of the group B two of five tested CFUs and in the area of group A all five CFUs showed these high MIC values. With filter pumps (Fig. 2b) *E. coli* with MICs of > 8 mg/L were already selected on day 34 to 42. In the period after treatment of the group A the same results were received.

### Excretion of antimicrobial active metabolites and its transfer into environment (experiment II)

For a comparison of biliary and renal elimination rates of the drug under different doses regimes and in order to investigate the input of drug residues in aerosol and dust of the stable via excrements of treated animals a pharmacokinetic study was performed after i.m. application of 0.3, 1, and 3 mg/kg ceftiofur. In addition to that, ceftiofur (3 mg/kg) were given per os to simulate oral uptake of active drug residues via the air of the stable.

#### Validation of analytical parameters

The development of the MRM-assay included the determination of major analytical parameters for DFC quantification in different matrices. Thereby LOD and LLOQ were determined in respective artificial matrices by extrapolation after adding ceftiofur (1 ng/mL) and subsequent sample preparation as described in the experimental section. The LOD for the quantification of DFC was calculated to be in the low ng/mL-range (Additional file 1: Analytical validation, Table S1).

Linearity of method for the matrix matches calibrations was tested for a concentration range of 1–500 ng/mL with acceptable regression values as depicted in the Additional file 1: Analytical validation, Figure S1. The coefficients of variation (CV) for the intra-assay imprecision including sample preparation of DFC from investigated sample matrices were determined using two different concentration levels (5 ng/mL and 100 ng/mL; *n* = 5 each). As Additional file 1: Table S2 (Analytical validation) illustrates obtained CV’s were in all matrices below 10 %, indicating a sufficient sample cleanup.

### Plasma

Plasma concentrations of DFC depended on dosage and treatment day (see Additional file 2). Thus DFC-concentrations in plasma were lower after treatment with 0.3 or 1 mg/kg in comparison to 3 mg/kg (i.m.). The highest amounts of 13.53 μg DFC/mL and 17.28 μg DFC/mL were observed 1 h after the first and third intramuscularly injection of 3 mg/kg ceftiofur.

The low plasma concentrations of DFC and metabolites (less than 0.1 μg/mL) after the oral treatment showed a minor absorption rate via the intestinal tract due to the chemical properties of ceftiofur.

### Renal excretion

The elimination studies after intramuscular treatment of swine with 0.3, 1, and 3 mg/kg ceftiofur showed that the majority of the administered ceftiofur dose was excreted via urine (see Additional file 3). Because of the different drinking pattern of each animal the amount of creatinine was measured and the concentration of DFC and DFC-metabolites were normalized to these values. It is obvious from the data, that the majority of ceftiofur is eliminated during the treatment period and only minor amounts are excreted afterwards. The low concentrations of DFC in urine after oral application reflected the poor oral absorption of ceftiofur and confirm the results examined in plasma.

### Biliary excretion

Since ceftiofur and its metabolites are eliminated primarily by renal excretion low amounts were quantified in faeces (see Additional file 4). The highest concentrations were measured on day 2 and 3. On day 3 262.94 ng DFC/g faeces was detected after intramuscular treatment with 3 mg/kg ceftiofur. Corresponding to the former results the biliary elimination was clearly dose-dependent. The poor absorption rate of ceftiofur into the blood after per os treatment resulted in high concentrations of DFC and DFC-metabolites in faeces. The DFC-concentrations measured on day 2 and 3 after oral treatment were slightly higher than after intramuscularly application.

### Sedimentation dust

**Fig. 3:**
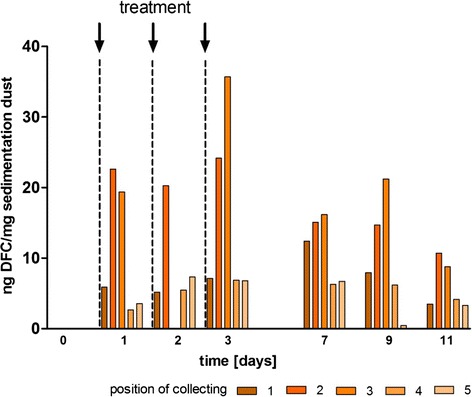
Concentrations of DFC in sedimentation dust after treatment of six animals with 3 mg Ceftiofur/kgb.w. i.m. (experiment II). Samples of the sedimentation dust were collected at different locations of the barn (position 1: window ledge, position 2: feeding trough, position 3: resting place, position 4: ground between the boxes, position 5: opposite site of the boxes for the animals). DFC-concentrations were analyzed by mass spectrometry. The arrows indicated the time points of treatment

Although ceftiofur was applicated by i.m. injection, residues of DFC and metabolites were present in the sedimentation dust samples (Fig. 3 and Additional file 5). Significant levels of DFC were detected after intramuscularly treatment with 3 mg/kg and 1 mg/kg with up to 61.22 ng DFC/mg dust (Additional file 5). During the application period the values of DFC in dust increased. After treatment DFC-values decreased but were still detectable until day 11 in every sample of dust. The level of measured DFC-amounts depended on the collecting position. Samples from the positions next to the box of the animals (position 2 and 3) showed the highest amounts. Lower concentrations of DFC were detected in the dust at the ground in front of the box (position 4) due to the presence of feed residues, which accumulated in this area.

After the per os treatment lower amounts of DFC with up to 4.86 ng DFC/mg were quantified in dust.

### Aerosol

**Fig. 4:**
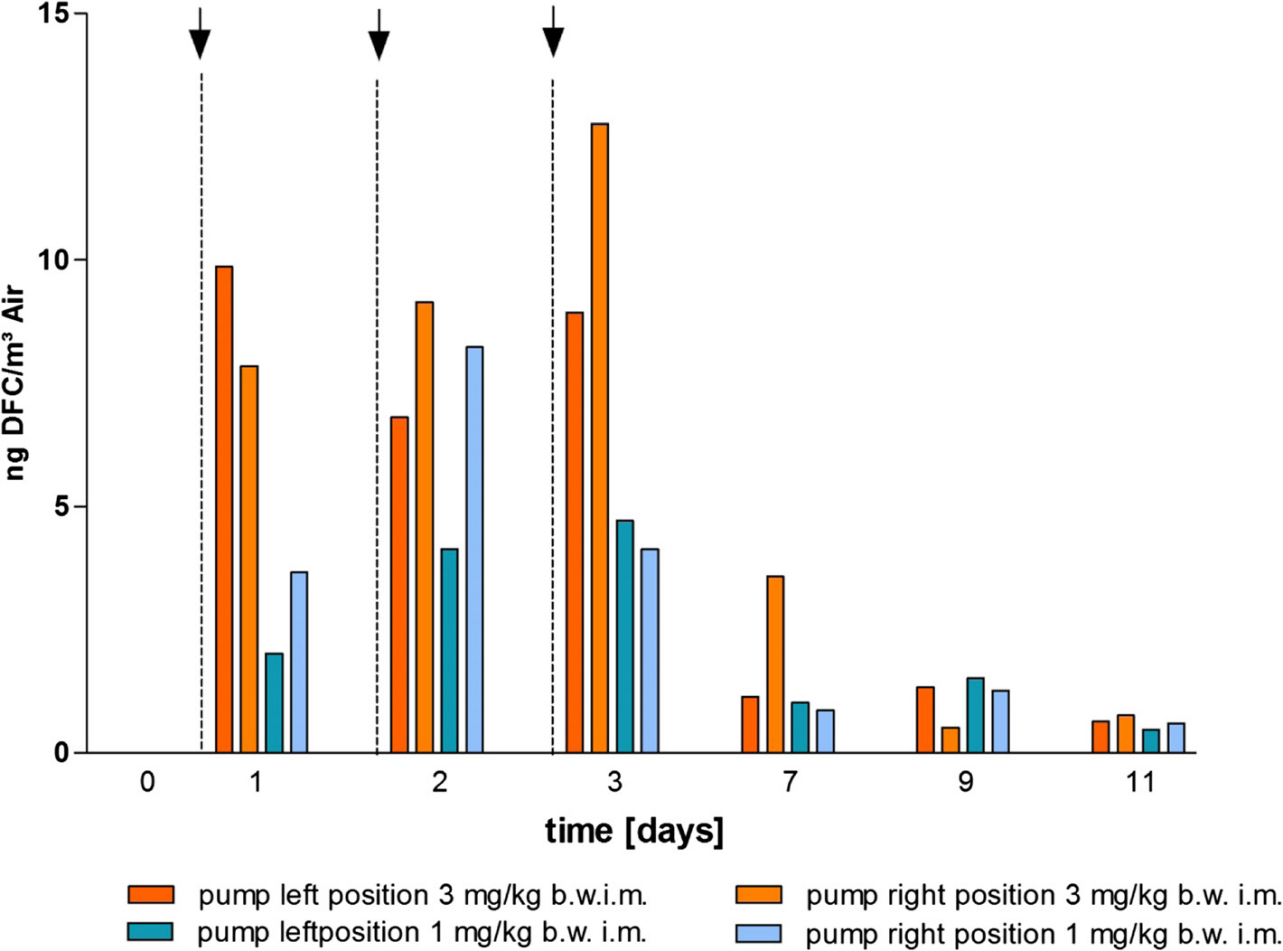
Concentrations of DFC in the aerosol of the stable after treatment of six animals with 3 mg Ceftiofur/kg b.w. i.m. or 1 mg/kg b.w. i.m. (experiment II). Via air pumps located near to the animals at the feeding through and on the left side of the resisting place probes of the aerosol were sampled and the DFC content measured by mass spectrometry. For determination of the amount of the dust in the aerosol the filters were weighted before and after the 8 h sampling period

In all filter pump collected aerosol samples residues of DFC and metabolites were detectable (Fig. 4 and Additional file 6). Depending on the dosage, the highest amounts were measured after intramuscular treatment with 3 mg ceftiofur/kg. On day 3 values up to 12.8 ng DFC/m^3^ air were quantified. After treatment with the lower dosages (1 and 0.3 mg/kg i.m.) decreased amounts of DFC in the aerosol samples were examined. As already shown very low amounts of DFC were detectable in the aerosol samples of the stable after oral treatment. Furthermore no residues were measurable in the days after per os treatment.

## Discussion

The study shows that treatment according to license of a group of animals in a stable with the antimicrobial ceftiofur leads to an alteration of the resistance pattern of the animals. Although administered by injection, active drug metabolites were found in dust and aerosol of the stable and also ESBL-carrying *E. coli* were detected in the aerosol. But the key finding of the study was that not only the treated animals from group B showed ESBL resistant *E. coli* after the second application of ceftiofur. For these ESBL resistant *E. coli* were also present in the untreated animals of group A, whom were housed in the same stable, during their first treatment. Therefore it is indicated that either drug residues in the air of the stable or ESBL resistant *E. coli* originated from the treated animals are the trigger for the resistance pattern of initially untreated animals.

At day 34 of the experiment the first *E. coli* isolates with MIC > Ecoff were detected in the group of treated animals and later on all animals were affected. In this context it should be noted that at the beginning of the experiment the stable as well as all animals were *E. coli* ESBL negative. Therefore, only two treatments with ceftiofur according to the license were sufficient to induce a shift of the MIC of *E. coli* in these animals.

The occurrence of resistant *E. coli* bacteria under ceftiofur treatment can be explained by spontaneous mutations of β-lactamase encoding genes or by acquisition of resistance encoding genes from other bacteria of the gut. The lactamases TEM-7, TEM-12 and SHV-2 are examples for lactamases which obtain the capability to inhibit cephalosporins of the third generation as a result of a change of one codon in the DNA [36]. At the beginning of the experiment the microbiome of the animals was examined only for resistant *E. coli* due to the use of endo-agar. Thus, presence of other bacteria, e.g. non-*E. coli* microbes, which carried ESBL-encoding genes cannot be excluded. ESBL of the CTX-M family were found in this study (data not shown). For this enzyme family it is unlikely to be building up through single amino acid alterations [37]. These lactamase encoding genes are plasmid-mediated [38, 39] and exchanged by conjugation. A study of Frye et al. [13] showed that most of ß-lactam resistance genes were shared by different bacteria as demonstrated for *E. coli* and *Salmonella* species. The presence of plasmid mediated CTX-M ESBLs in this study is a strong indication for acquisition of resistance genes by horizontal transfer from other bacteria of the microbiome in the treated animals.

Although ceftiofur was administered by intramuscular injection, DFC as antimicrobial active metabolite was found in the dust and aerosol of the stable. The only way for the entry of these antimicrobial residues into the environment of the stable was via urine and faeces. Therefore concentration – time profiles of DFC were measured in urine and faeces of the treated animals. To our knowledge there are no data about the composition of metabolites in faeces after a treatment with ceftiofur. But in urine additional to the parental ceftiofur (28.3 %) the major metabolites of ceftiofur are the DFC-dimer (27,1 %) and DFC-cystein (32,3 %) [27]. All these metabolites retained their intact β-lactam ring and can be reduced back to DFC. Therefore about 88 % of the applied ceftiofur dose is excreted in an antimicrobial active form [40]. In a study by Gilbertson [41] microbiological activity for several days was found in urine samples. Consequently drug residues were measured in sedimentation dust and aerosols after detection of DFC and its metabolites in urine and faeces. Based on the data of this study, the primary route of drug entry into the vicinity of ceftiofur treated animals seems to be via the urine.

Although a fast degradation of ceftiofur and its metabolites in faeces and urine was described [41] and it was assumed that this drug is inactivated by the microbiota of the colon during the biliary secretion [28], we could show the entry and persistence of DFC and its metabolites in the environment. Due to the presence of the intact β-lactam-ring the detected metabolites retains microbiological activivity [31]. It cannot be ruled out that the top layer of the dust is vulnerable to photodegradation and thus also protecting the underlying layers, which could be a mechanism for conservation of microbiological activity of the drug residues in these underlying layers [41]. In a study by Hamscher et al. [42] antibiotic residues could be detected in 20 years old dust samples from a stable. In the case of insufficient cleaning of an animal stable after the treatment with antimicrobials potential residuals could be persist for a long time. After treatment with 3 and 1 mg/kg ceftiofur (i.m.) the highest amounts of the drug were found in dust. Higher DFC-concentrations in dust after application of 1 mg/kg ceftiofur in comparison to the 3 mg/kg dosage can be explained by an increased activity of the animals because the pigs were in growth during the experiment and the lower dosage was tested afterwards. Due to the increased activity of the older pigs more dried urine and feaces are swirled up in the stable and leads to a higher DFC-concentration in the aerosol.

Parallel to the detection of active DFC-metabolites in dust and aerosol ESBL producing *E. coli* in the air of the stable were identified. Moreover, the animals of the formerly untreated group (group A) show clinically resistant *E. coli* bacteria during their first treatment on day 45–47. These results indicate that either low amounts of the drug in the stable or occurrence of *E. coli* carrying the ESBL resistance from previously treated animals (group B) in the air of the stable induce a MIC shift of the *E. coli* from the untreated animals towards clinical resistance. This implies that only two consecutive treatments with ceftiofur were necessary to induce a resistant phenotype in untreated animals housed in the same barn as the treated animals.

The carry-over of antimicrobials like ceftiofur and their active metabolites in the stable could facilitate the development of antibacterial resistance due to ingestion by untreated animals. These very low concentrations of the antimicrobial in the gut of untreated animals could promote a selection advantage to bacteria in which a few point mutations within the genes give rise to the extended spectrum phenotype [43]. A study performed by Zessel [23] demonstrated that an oral medication of some pigs with sulphadiazine resulted in a residual level in the environment that cause detectable amounts of this antibiotic in the plasma of untreated pigs that were kept in the same stable. On days 0, 3, and 4 of the experiment plasma samples of the untreated control group were taken and the potential amount of DFC and its metabolites was determined. However, no residues could be detected in plasma or were below the detection limit of the method. But the lack of evidence of absorption of ceftiofur into the blood plasma does not argue against the possibility that these sub-therapeutic doses may have contributed to the development of resistant *E. coli* in the untreated animals.

Detection of ESBLs of the CTX-M family in samples of the control group may (group A) argue for the transfer of plasmids or an exchange of resistant bacteria between the two animal groups and could be also a reason for the resistance of untreated animals. By using of group-owned stable equipment and subsequent feeding the animals of control group and the animals of the treated group, the exchange of bacteria between both groups via the involved personal or equipment was minimized. Therefore it can be assumed that the transmission of bacteria occurred by air or aerosols. Different studies [44–46] showed the transition of gram negative bacteria, mainly *E. coli,* in the air. In our study *E. coli* were recovered from the environment by use of air pumps and endo-agar plates to determine the MIC. Within the first days of the study only a few CFU were isolated. Higher room temperatures in combination with lower humidity in the stable may be an explanation for this finding. Because of the age of the swine the stable had to be heated to 24.5 °C in the first two weeks. Mueller and Dinter [47] confirmed these both parameters as factors influencing the tenacity of *E. coli*. Furthermore the increased number of bacteria in the air of the stable during the experiment can be a reason for a better recovery as well. Zucker and Mueller [44] reported that faeces are an origin of bacteria in the air. This is particularly significant when it is assumed that during treatment with ceftiofur bacteria of the intestinal microbiota acquired resistance and were spread after excretion in the air inside the barn. *E. coli* in the air with MIC > 64 mg/L in both areas of the stable were detected. Therefore it can be assumed that an exchange of bacteria occurred between the two groups. The uptake of resistant bacteria from the environment and integration into the gut microbiome may be a conceivable explanation for the development of resistant *E. coli* in the control group.

Without the presence of antibiotics, resistant bacteria have no advantage in comparison to the wild type. A general opinion is that through the acquisition of resistance genes bacteria are subjected to a fitness loss and were displaced by the wild type [48]. But the study of Jiang and co-workers [49] showed the persistence of resistant bacteria in the microbiome of animals for a long time after a treatment and demonstrated the possible integration of resistant bacteria into the microbiome without a fitness loss. It cannot be ruled out that very low concentrations of DFC in the gut, ingested from the environment, supported the integration of these resistant bacteria into the microbiome of the untreated animals without a fitness loss. The impact on the intestinal microbiota by the use of ceftiofur to these pre-stressed animals is obvious by comparison of the amounts of detected CFUs with MICs > 64 mg/L between the two animal groups. A statistically significant difference (*p* < 0.05) was observed between MICs of the untreated animals after application of ceftiofur and the results from treated animals after its first and second treatment. Subsequent to the first use of ceftiofur no CFU with increased MICs were detected in samples of the treated group (group B) with the microdilution technique because no resistant *E. coli* was part of the microbiome. During the experiment *E.coli* of the gut acquired resistance encoding genes and CFUs with increased MICs were found following a second treatment. The intestinal microbiota of the animals of the untreated group (group A) harboured resistant *E. coli* at this time, but these were only detectable after enrichment. Under the selective pressure of ceftiofur and their active metabolites in the gut during the treatment of the control group (group A, day 45 to 47), such bacteria with ESBL encoding genes have a selective advantage in growth while susceptible bacteria will be inhibited. This growth benefit may be an explanation for the high amounts of detected resistant CFUs.

## Conclusions

In conclusion, the use of ceftiofur in livestock leads to residues of DFC and other active metabolites in the environment which are distributed through the whole stable and influence the bacteria of the stable or can be incorporated by other animals of the livestock. In this study it was shown that the treatment of several animals has an influence on the resistance situation of all individuals in the barn. Thus, a prudent use of antimicrobials in a combination with good hygienic conditions is required and further studies are necessary to investigate the role of intestinal *E. coli* as a possible reservoir of antimicrobial resistance.

## Additional files

Additional file 1:
**Analytical validation. Table S1.** Calculated limits of detection (LOD) and lower limits of quantification (LLOQ) for different sample matrices for quantification of DFC using UPLC-MS/MS. **Figure S1.** Calibration curve for the quantification of DFC from plasma. Table inset shows regression coefficients for the quantification from different sample matrices. Linearity of method was tested for concentration range of 1–500 ng/ml. **Table S2.** Coefficients of variation (CV) at two different concentrations for the analysis of DFC from investigated sample matrices (*n* = 5). (PPTX 108 kb) Additional file 2:
**Concentrations of DFC in plasma after application of diverse dosages of ceftiofur i.m. (3 mg/kg b.w.; 1 mg/kg b.w. and 0.3 mg/kg b.w.) and p.o. (3 mg/kg b.w.).** (DOCX 15 kb) Additional file 3:
**Concentrations of DFC in urine after application of diverse dosages of ceftiofur i.m. (3 mg/kg b.w.; 1 mg/kg b.w. and 0.3 mg/kg b.w.) and p.o. (3 mg/kg b.w.).** (DOCX 15 kb) Additional file 4:
**Concentrations of DFC in feces after application of diverse dosages of ceftiofur i.m. (3 mg/kg b.w.; 1 mg/kg b.w. and 0.3 mg/kg b.w.) and p.o. (3 mg/kg b.w.).** (DOCX 14 kb) Additional file 5:
**Concentrations of DFC in the dust of the stable after application of diverse dosages of ceftiofur i.m. (3 mg/kg b.w.; 1 mg/kg b.w. and 0.3 mg/kg b.w.) and p.o. (3 mg/kg b.w.).** (DOCX 17 kb) Additional file 6:
**Concentrations of DFC in the aerosol (sampled by air pumps) after application of diverse dosages of ceftiofur i.m. (3 mg/kg b.w.; 1 mg/kg b.w. and 0.3 mg/kg b.w.) and p.o. (3 mg/kg b.w.).** (DOCX 16 kb)
