# Supplementary material for: Effects of ceftiofur treatment on the susceptibility of commensal porcine E.coli – comparison between treated and untreated animals housed in the same stable
Source: BMC Vet Res. 2015 Oct 15;11:265. doi: 10.1186/s12917-015-0578-3 (PMC4608134; doi:10.1186/s12917-015-0578-3)
Supplement: Additional file 1: — Analytical validation. Table S1. Calculated limits of detection (LOD) and lower limits of quantification (LLOQ) for different sample matrices for quantification of DFC using UPLC-MS/MS. Figure S1. Calibration curve for the quantification of DFC from plasma. Table inset shows regression coefficients for the quantification from different sample matrices. Linearity of method was tested for concentration range of 1–500 ng/ml. Table S2. Coefficients of variation (CV) at two different concentrations for the analysis of DFC from investigated sample matrices (n = 5). (PPTX 108 kb) [file 12917_2015_578_MOESM1_ESM.pptx]

## Slide 1
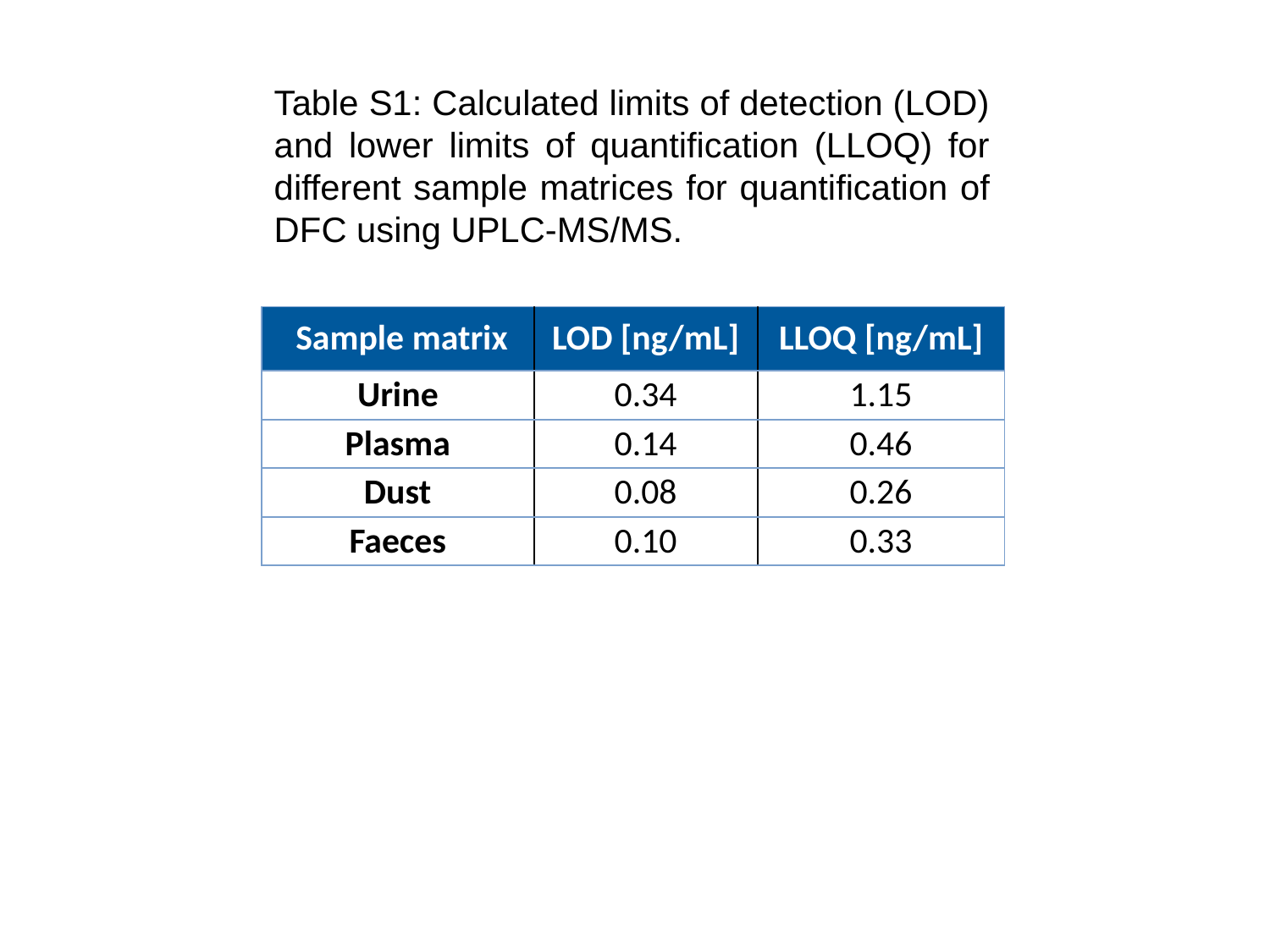

Table S1: Calculated limits of detection (LOD) and lower limits of quantification (LLOQ) for different sample matrices for quantification of DFC using UPLC-MS/MS.
| Sample matrix | LOD [ng/mL] | LLOQ [ng/mL] |
| --- | --- | --- |
| Urine | 0.34 | 1.15 |
| Plasma | 0.14 | 0.46 |
| Dust | 0.08 | 0.26 |
| Faeces | 0.10 | 0.33 |

## Slide 2
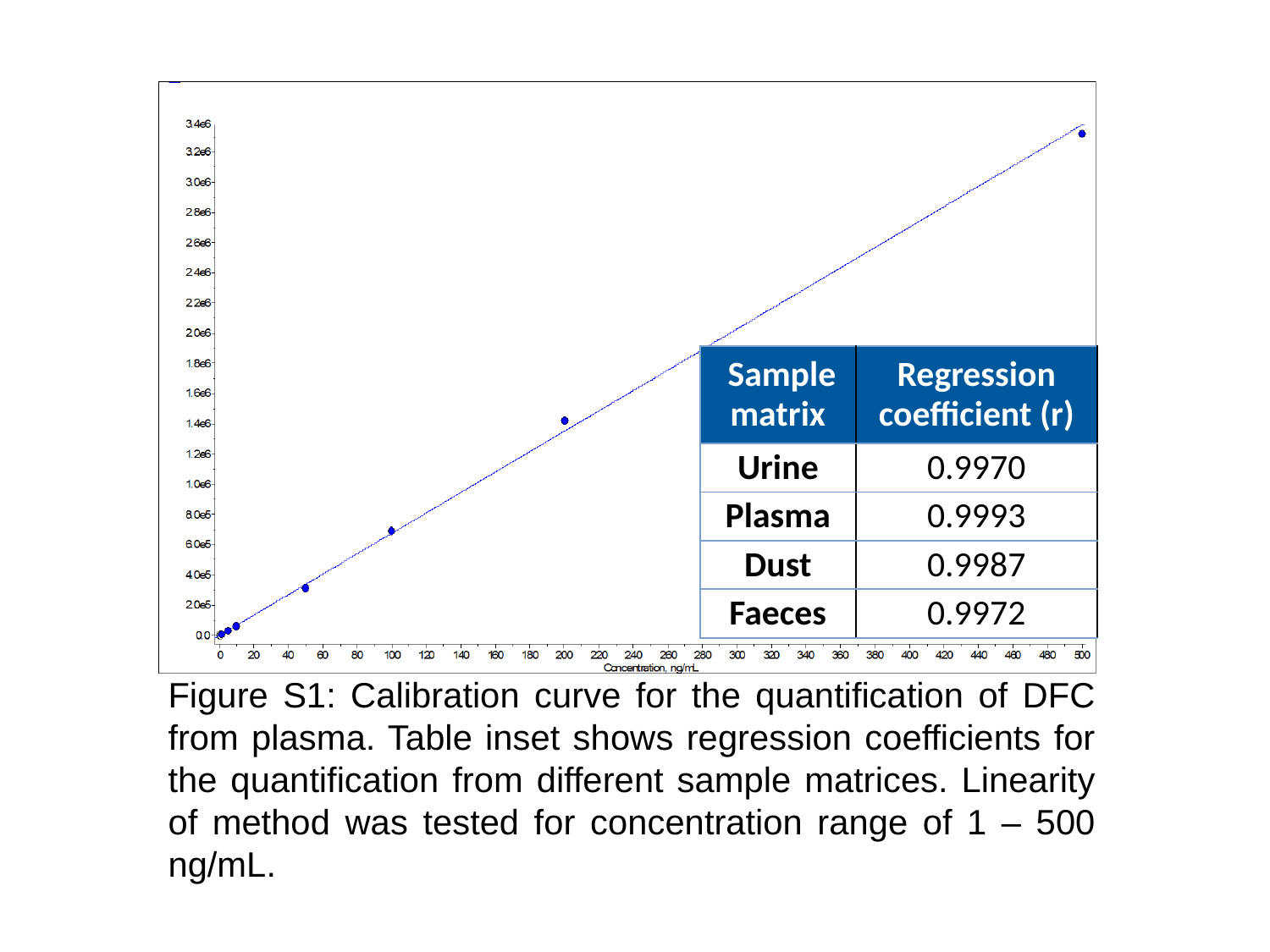

| Sample matrix | Regression coefficient (r) |
| --- | --- |
| Urine | 0.9970 |
| Plasma | 0.9993 |
| Dust | 0.9987 |
| Faeces | 0.9972 |
Figure S1: Calibration curve for the quantification of DFC from plasma. Table inset shows regression coefficients for the quantification from different sample matrices. Linearity of method was tested for concentration range of 1 – 500 ng/mL.

## Slide 3
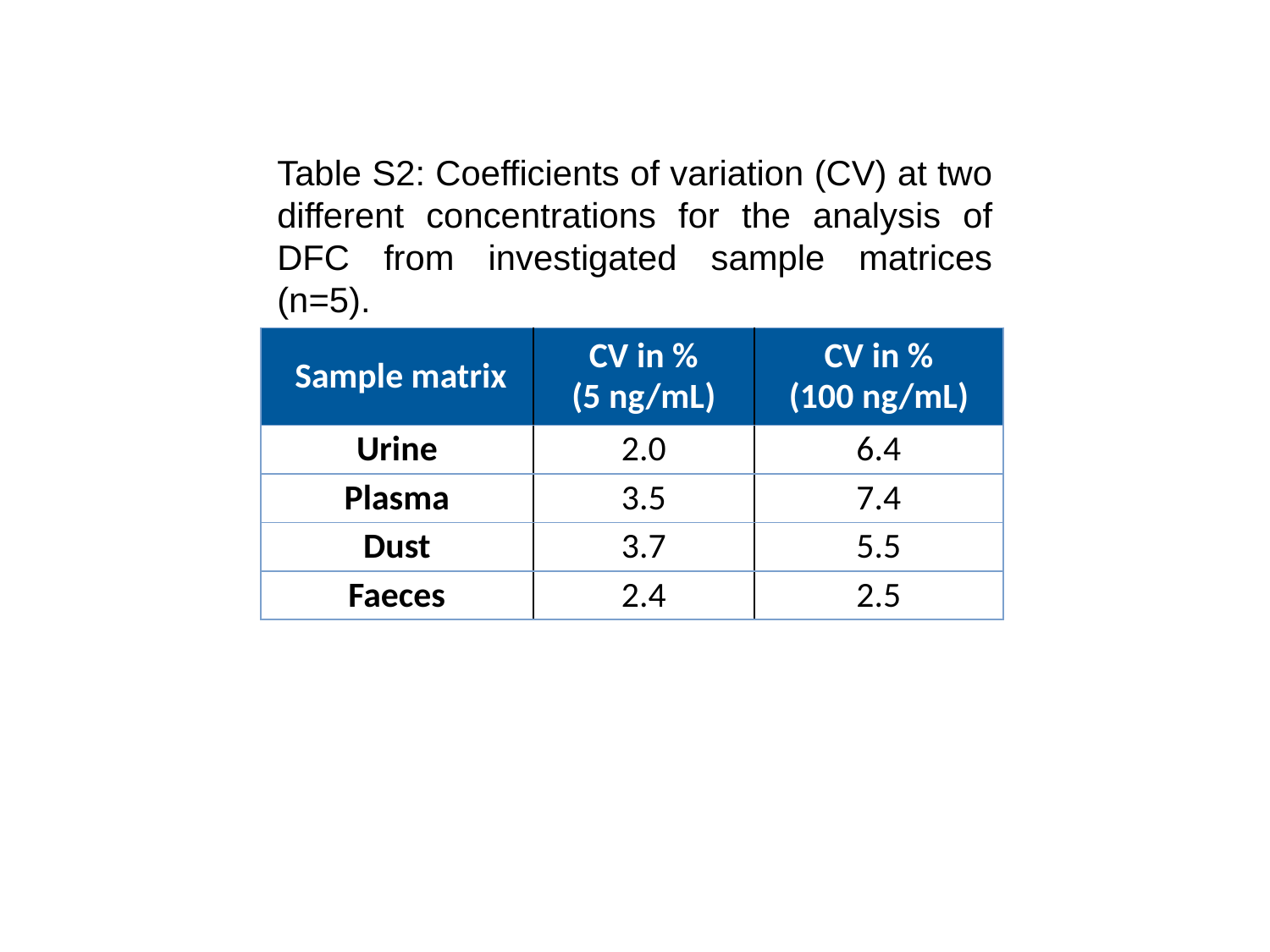

Table S2: Coefficients of variation (CV) at two different concentrations for the analysis of DFC from investigated sample matrices (n=5).
| Sample matrix | CV in % (5 ng/mL) | CV in % (100 ng/mL) |
| --- | --- | --- |
| Urine | 2.0 | 6.4 |
| Plasma | 3.5 | 7.4 |
| Dust | 3.7 | 5.5 |
| Faeces | 2.4 | 2.5 |
