# Supplementary material for: Effects of ceftiofur treatment on the susceptibility of commensal porcine E.coli – comparison between treated and untreated animals housed in the same stable
Source: BMC Vet Res. 2015 Oct 15;11:265. doi: 10.1186/s12917-015-0578-3 (PMC4608134; doi:10.1186/s12917-015-0578-3)
Supplement: Additional file 2: — Concentrations of DFC in plasma after application of diverse dosages of ceftiofur i.m. (3 mg/kg b.w.; 1 mg/kg b.w. and 0.3 mg/kg b.w.) and p.o. (3 mg/kg b.w.). (DOCX 15 kb) [file 12917_2015_578_MOESM2_ESM.docx]

|  | Concentration of Desfuroylceftiofur and DFC-metabolites | | | | | | | | |
| --- | --- | --- | --- | --- | --- | --- | --- | --- | --- |
|  | [µg DFC/mL plasma] (mean + SD) | | | | | | | | |
| sampling time [h] | 3 mg/kg b.w. i.m. | | 1 mg/kg b.w. i.m. | | 0.3 mg/kg b.w. i.m. | | sampling time [h] | 3 mg/kg b.w. p.o. | |
| 0 | 0,11 | ± 0.044 | n.d. | | 0,05 | ± 0.06 | 0 | 0,02 | ± 0.01 |
| 1 | 13,53 | ± 2.20 | n.d. | | 1,37 | ± 0.53 | 2 | 0,06 | ±0.02 |
| 6 | 9,45 | ± 0.93 | 2,53 | ± 0.39 | 1,17 | ± 0.52 | 4 | 0,08 | ± 0.03 |
| 24 | 2,75 | ± 0.41 | 0,61 | ± 0.17 | 0,17 | ± 0.04 | 6 | 0,08 | ± 0.03 |
| 48 | 3,21 | ± 0.47 | 0,97 | ± 0.34 | 0,33 | ± 0.33 | 24 | 0,04 | ± 0.01 |
| 49 | 17,28 | ± 2.44 | 4,80 | ± 1.01 | 1,08 | ± 0.38 | 48 | 0,03 | ± 0.00 |
| 54 | 10,57 | ± 1.40 | 3,02 | ± 0.59 | 0,64 | ± 0.19 | 50 | 0,08 | ± 0.03 |
| 71 | 1,59 | ± 0.91 | 0,81 | ± 0.26 | 0,17 | ± 0.07 | 52 | 0,08 | ± 0.03 |
| 96 | 2,88 | ± 1.05 | 0,35 | ± 0.15 | 0,08 | ± 0.03 | 54 | 0,08 | ± 0.03 |
|  |  |  |  |  |  |  |  |  |  |

Additional file 2:
