# Supplementary material for: Effects of ceftiofur treatment on the susceptibility of commensal porcine E.coli – comparison between treated and untreated animals housed in the same stable
Source: BMC Vet Res. 2015 Oct 15;11:265. doi: 10.1186/s12917-015-0578-3 (PMC4608134; doi:10.1186/s12917-015-0578-3)
Supplement: Additional file 3: — Concentrations of DFC in urine after application of diverse dosages of ceftiofur i.m. (3 mg/kg b.w.; 1 mg/kg b.w. and 0.3 mg/kg b.w.) and p.o. (3 mg/kg b.w.). (DOCX 15 kb) [file 12917_2015_578_MOESM3_ESM.docx]

|  |  |  |  |  |  |  |  |  |  |
| --- | --- | --- | --- | --- | --- | --- | --- | --- | --- |
|  | Concentration of Desfuroylceftiofur and DFC-metabolites normalized to creatinine | | | | | | | | |
|  | [µg DFC/mL urine] (mean + SD) | | | | | | | | |
| sampling day |  | 3 mg/kg b.w. i.m. | | 1 mg/kg b.w. i.m. | | 0.3 mg/kg b.w. i.m. | | 3 mg/kg b.w. p.o. | |
| day 0 |  | n.d. | | n.d. | | n.d. | | n.d. | |
| day 1 |  | 22,28 | ± 25.36 | 1,61 | ± 0.32 | 0,22 | ± 0.05 | 0,10 | ± 0.06 |
| day 2 |  | 15,75 | ± 17.61 | 3,30 | ± 2.07 | 0,39 | ± 0.07 | 0,10 | ± 0.07 |
| day 3 |  | 9,96 | ± 2.56 | 4,89 | ± 1.71 | 0,55 | ± 0.28 | 0,05 | ± 0.02 |
| day 7 |  | 0,09 | ± 0.04 | 0,06 | ± 0.05 | 0,01 | ± 0.00 | n.d. | |
| day 9 |  | 0,05 | ± 0.04 | 0,02 | ± 0.02 | 0,01 | ± 0.00 | 0,35 | ± 0.73 |
| day 11 |  | 0,02 | ± 0.01 | 0,01 | ± 0.01 | n.d. | | n.d. | |
|  |  |  |  |  |  |  |  |  |  |

Additional file 3:
