# Supplementary material for: Effects of ceftiofur treatment on the susceptibility of commensal porcine E.coli – comparison between treated and untreated animals housed in the same stable
Source: BMC Vet Res. 2015 Oct 15;11:265. doi: 10.1186/s12917-015-0578-3 (PMC4608134; doi:10.1186/s12917-015-0578-3)
Supplement: Additional file 4: — Concentrations of DFC in feces after application of diverse dosages of ceftiofur i.m. (3 mg/kg b.w.; 1 mg/kg b.w. and 0.3 mg/kg b.w.) and p.o. (3 mg/kg b.w.). (DOCX 14 kb) [file 12917_2015_578_MOESM4_ESM.docx]

|  | Concentration of Desfuroylceftiofur and DFC-metabolites in faeces | | | | | | | | |
| --- | --- | --- | --- | --- | --- | --- | --- | --- | --- |
|  | [ng DFC/g faeces] (mean + SD) | | | | | | | | |
| sampling day |  | 3 mg/kg b.w. i.m. | | 1 mg/kg b.w. i.m. | | 0.3 mg/kg b.w. i.m. | | 3 mg/kg b.w. p.o. | |
| day 0 |  | n.d. | | n.d | | n.d. | | n.d. | |
| day 1 |  | 7,20 | ± 7.38 | n.d. | | 4,78 | ± 2.38 | n.d. | |
| day 2 |  | 234,56 | ± 86.55 | 26,42 | ± 8.07 | 8,47 | ± 4.47 | 581,33 | ± 510.08 |
| day 3 |  | 262,94 | ± 209.72 | 61,79 | ± 20.43 | 11,35 | ± 2.01 | 265,65 | ± 311.75 |
| day 7 |  | 6,70 | ± 13.70 | 5,33 | ± 0.45 | 6,67 | ± 1.49 | 9,55 | ± 4.39 |
| day 9 |  | n.d. | | n.d. | | n.d. | | n.d. | |
|  |  |  |  |  |  |  |  |  |  |

Additional file 4:
