# Supplementary material for: Effects of ceftiofur treatment on the susceptibility of commensal porcine E.coli – comparison between treated and untreated animals housed in the same stable
Source: BMC Vet Res. 2015 Oct 15;11:265. doi: 10.1186/s12917-015-0578-3 (PMC4608134; doi:10.1186/s12917-015-0578-3)
Supplement: Additional file 5: — Concentrations of DFC in the dust of the stable after application of diverse dosages of ceftiofur i.m. (3 mg/kg b.w.; 1 mg/kg b.w. and 0.3 mg/kg b.w.) and p.o. (3 mg/kg b.w.). (DOCX 17 kb) [file 12917_2015_578_MOESM5_ESM.docx]

Additional file 5:
